# The Association Between Refractive Errors and Cataract: The Tehran Eye Study

**DOI:** 10.4103/0974-9233.80705

**Published:** 2011

**Authors:** Hassan Hashemi, Mehdi KhabazKhoob, Mohammad Miraftab, Kazem Mohammad, Akbar Fotouhi

**Affiliations:** Farabi Eye Hospital, Tehran University of Medical Sciences, Tehran, Iran; 1 Noor Ophthalmology Research Center, Noor Eye Hospital, Tehran, Iran; 2 Department of Epidemiology and Biostatistics, School of Public Health, Tehran University of Medical Sciences, Tehran, Iran

**Keywords:** Cataract, Population-Based Study, Refractive Errors

## Abstract

**Purpose:**

To determine the association between refractive errors and different types of cataract in Tehran, Iran.

**Materials and Methods:**

In a cross-sectional survey with a stratified cluster sampling approach, refractive errors were tested under cycloplegia. Myopia and hyperopia were defined as a spherical equivalent refractive error <-0.5 diopters (D) and more than +0.5 D, respectively. Cataract was graded according to the Lens Opacities Classification System III classification and the association between refractive errors and cataract was assessed. Of 1434 participants over the age of 40 years who participated in the study, data from 1313 right eyes were analyzed.

**Results:**

The mean age of the participants was 52.7 ± 10.0 years, and 58.3% (*n* = 767) were female. Overall, myopia was more prevalent among those with cataract (odds ratio [OR]: 2.00, 95% confidence interval [CI]: 1.38–2.89). Based on the type of cataract and refractive errors, the odds of myopia was significantly higher with nuclear cataracts (OR: 1.81, 95% CI: 1.14–2.87). The odds of myopia was higher for cases of nuclear cataract with some degrees of posterior subcapsular cataract (PSC) (OR: 3.33, 95% CI: 1.42–7.80). Of nine participants with cortical cataract, seven participants had hyperopia (OR: 3.77, 95% CI: 0.78–18.31).

**Conclusion:**

Individuals with nuclear and PSC showed a significantly higher prevalence of myopia while the prevalence of hyperopia was lower in those with cataract. High myopia was seen in higher grades of nuclear cataract. The high percentage of hyperopia was also significant in patients with cortical cataract. More studies are necessary to clarify the correlation between cortical cataract and hyperopia.

## INTRODUCTION

Refractive errors after cataract have been previously examined in population-based studies.^1^–^6^ Myopia is the most frequent refractive error after cataract surgery according to extensive studies such as the Blue Mountains Eye study and Reykjavik Study.^4^,^7^ Based on reports by Mitchell,^3^ Chang,^8^ Fotedar^9^ and Wong,^6^ nuclear cataract is the most common cause of myopic shift. Epidemiologically and biologically, the relationship between other types of cataract and refractive errors is still not clearly understood. While many studies have confirmed the relationship between nuclear cataract and myopia, few have reported a hyperopic shift or even astigmatism after cortical cataract.^10^,^11^ The majority cases of cataract occur after the onset of presbyopia. Hence, presbyopia and refraction changes due to cataract can affect the visual acuity and visual quality. As the pattern of refractive errors in different age groups is already defined, identifying the exact correlation between cataract and refractive errors can assist in the prediction of the possible refractive errors, which will help the control refraction from the very beginning stages of the development of cataract. Apart from the correlation between cataract and myopia, further studies need to be conducted on the correlation between types of cataract and the type of refractive errors. Further studies on the relationship between cataract and refractive errors will allow comparison with previous findings and help to elucidate unknown association. Through a population-based study, the Tehran Eye Study, we have reported the prevalence of refractive errors and lens opacity.^12^,^13^ In this study, we report the effects of different factors, including ethnicity and biometry, on different populations and the correlation between nuclear cataract and myopia in addition to the correlation between other refractive errors and the type of cataract.

## MATERIALS AND METHODS

The Tehran Eye Study is a cross-sectional survey that was conducted in 2002 in Tehran.^14^ The methodology of the Tehran Eye Study has been published.^14^ Briefly, samples were selected through a stratified cluster sampling approach with respect to the population of each district in Tehran. In each cluster, 10 households were approached in a systematic clockwise fashion and their members were invited for ophthalmic examinations. Participants were transferred to an eye clinic free of charge and had complete eye examinations, including tests of corrected and uncorrected visual acuity, manifest and cycloplegic refraction, intraocular pressure, slit lamp and retinoscopy. Participants’ spectacles were checked with a lensometer. In addition, the participants were interviewed regarding demographics, history of eye disease or trauma, diabetes, hypertension and previous eye examinations.

### DEFINITIONS

Upon examination, the degree of lens opacity was graded according to the Lens Opacities Classification System (LOCS III) grading guidelines. A gradable cataract was defined as LOCS III grade 3 or more in C and/or N and/or a grade 2 or more posterior subcapsular cataract (PSC).

A spherical-equivalent refraction equal to or more than -0.50 diopter (D) was defined as myopia. The cut point for hyperopia was considered to be +0.50 D and the range between -0.49 and +0.49 D was defined as normal. Because of the high correlation between the refractive errors of the right and left eyes, only data from the right eyes were analyzed. Therefore, of 1434 participants over the age of 40 years, 57 people who had a history of cataract eye surgery in the right eye and 64 people with missing data for type of cataract and refractive errors were excluded from this study, resulting in 1313 right eyes that were analyzed.

The prevalence rates of refractive errors based on the type of cataract were calculated in percentages. Correlations between different types of cataract and refractive errors were detected using logistic regression tests. The design effect of cluster sampling was considered when computing 95% confidence intervals (CI) and standard errors, and results were adjusted. *P* <0.05 was considered statistically significant.

The Research and Ethics Committee of Noor Ophthalmology Research Center and the Ethics Committee of the National Research Center for Medical Sciences approved the study. All participants were informed about the project and procedures in their native language and all consented to examination and inclusion in the study.

## RESULTS

The mean age of the participants was 52.7 ± 10.0 years, and 58.3% (*n* = 767) were female. Based on cycloplegic refraction, the prevalence of hyperopia and myopia was 57.30% (95% CI: 54.30–60.29) and 19.43% (95% CI: 17.06–21.80), respectively. Hyperopia significantly increased with age (*P* < 0.001) and myopia showed no significant correlation with age (*P* = 0.276). The prevalence of cataract was 16.86% (95% CI: 14.76–18.95).

Table 1 presents the percentages of refractive errors by type of cataract. Regardless of the type of cataract, the prevalence of myopia and hyperopia among subjects affected by cataract was 30.17% and 55.31%, respectively. Logistic regression tests showed that the prevalence of myopia was significantly higher among subjects with cataract, with an odds ratio (OR) of 2.00 (95% CI: 1.38–2.89) [Table 1]. In terms of cataract type, logistic regression revealed significantly higher rates of myopia among subjects with nuclear cataract and PSC and nuclear and PSC compared with those free of cataract.

**Table 1:** Prevalence of refractive errors for different types of cataract

	n	Myopia		Hyperopia	
		% (95% CI)	OR (95% CI)	% (95% CI)	OR (95% CI)
Non-cataract	1099	18.43 (15.98−20.88)	1	56 (52.87−59.14)	
Cataract					
Nuclear	124	27.52 (19−36.04)	1.81 (1.14−2.87)**	56.88 (47.43−66.33)	0.92 (0.61−1.39)
Cortical	9			77.78 (40.00−97.18)	3.77 (0.78−18.31)
Posterior subcapsular	28	38.10 (15.44−60.75)	2.85 (1.14−7.15)**	61.90 (39.25−84.56)	1.22 (0.49−3.04)
Nuclear and posterior subcapsular	33	43.48 (21.56−65.4)	3.33 (1.42−7.8)**	43.48 (21.56−65.4)	0.64 (0.27−1.48)
Nuclear and cortical	5	20.00 (0.50−71.64)*	1.12 (0.12−10.14)	20.00 (0.50−71.64)*	0.19 (0.02−1.70)
Cortical and posterior subcapsular	6	33.33 (4.32−77.72)*	3.00 (0.49−18.32)	33.33 (4.32−77.72)*	0.50 (0.08−3.06)
Nuclear and cortical and posterior subcapsular	9	33.33 (7.48−70.07)*	3.33 (0.73−15.15)	44.44 (13.70−78.80)*	1.04 (0.23−4.72)
All types	214	30.17 (23.37−36.96)	2.00 (1.38−2.89)**	55.31 (47.95−62.66)	0.66 (0.46−0.95)**

CI: Confidence interval, OR: Odds ratio,

* Statistically significant at the 5% level

** The 95% CI was calculated using binomial distribution

Based on the results, 77.78% of those with cortical cataract were also hyperopic. The prevalence of hyperopia was significantly lower in subjects with cataract.

**Figure 1:**
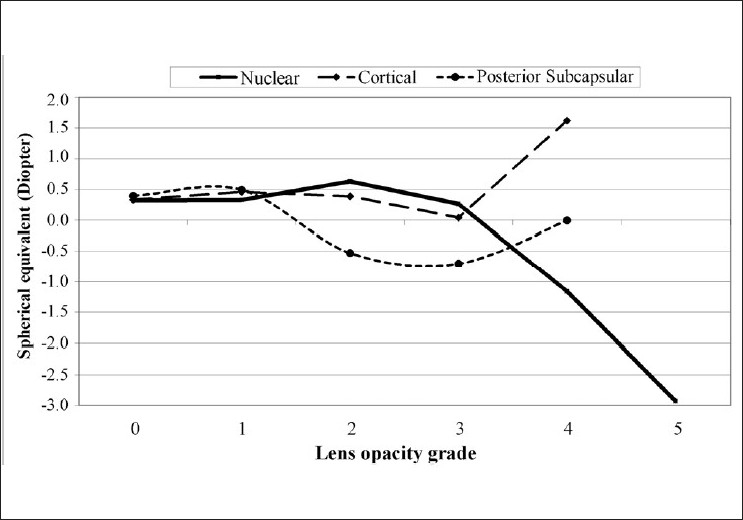
Spherical-equivalent refractive error by type and degree of cataract

Figure 1 presents the correlation between the degree of cataract and the spherical-equivalent refraction. The mean spherical equivalent in those with grade 1 cataract was 0.33 D, and there was a shift toward myopia as the grade of cataract increased; the mean spherical equivalent was -2.92 D with grade 5 cataract (*P* < 0.001). In cases of PSC, the spherical equivalent varied significantly with the grade of cataract (*P* = 0.002), but the change was not linear and refraction shifted toward emmetropia at higher grades of cataract (U-shaped). The mean spherical equivalent did not change significantly with different grades of cortical cataract (*P* = 0.869).

The results of refractive errors according to the grade of cataract are presented in Table 2. As shown, the risk of myopia significantly increased as the grade of nuclear and PSC cataract increased.

**Table 2:** Prevalence of refractive errors (percentage) by degree of lens opacity

LOCS III	Grade	Myopia		Hyperopia	
		% (95% CI)	OR (95% CI)	% (95% CI)	OR (95% CI)
Nuclear	0	19.20 (14.88−23.51)	1	52.94 (47.47−58.41)	1
	1	20.06 (15.86−24.25)	1.06 (0.72−1.54)	55.93 (50.74−61.13)	1.17 (0.87−1.56)
	2	16.45 (12.26−20.64)	0.83 (0.54−1.29)	60.86 (55.34−66.37)	1.42 (1.02−2.00)*
	3	24.17 (16.40−31.94)	1.36 (0.82−2.25)	57.5 (48.53−66.47)	1.15 (0.74−1.79)
	4	60.00 (36.48−83.52)	7.23 (2.69−19.43)*	40.00 (16.48−63.52)	0.53 (0.20−1.38)
	5	75.00 (19.41−99.36)**	13.34 (1.37−129.64)*	0	
Cortical	0	18.43 (15.61−21.26)	1	55.02 (51.4−58.65)	1
	1	20.98 (16.23−25.73)	1.12 (0.79−1.57)	58.39 (52.64−64.14)	1.23 (0.92−1.64)
	2	31.40 (21.39−41.40)	1.89 (1.14−3.14)*	58.14 (47.50−68.78)	1.21 (0.74−1.96)
	3	24.00 (6.01−41.99)	1.43 (0.55−3.73)	52.00 (30.95−73.05)	0.90 (0.42−1.96)
	4			100	
Posterior subcapsular	0	18.76 (16.20−21.31)	1	56.49 (53.25−59.74)	1
	1	20.83 (14.63−27.04)	1.19 (0.81−1.77)	55.36 (47.76−62.95)	0.96 (0.66−1.38)
	2	41.46 (25.72−57.21)	2.98 (1.59−5.6)*	51.22 (35.25−67.19)	0.85 (0.43−1.70)
	3	40.00 (11.92−68.08)	3.10 (0.94−10.25)	53.33 (24.74−81.93)	0.82 (0.29−2.33)

LOCS: Lens opacity classification system, CI: Confidence interval, OR: Odds ratio,

* Statistically significant at the 5% level,

** The 95% CI was calculated using binomial distribution

## DISCUSSION

The Tehran Eye Study is a comprehensive study on the population of Tehran, Iran, and details of our findings on refractive errors and lens opacity have already been published.^12^,^13^ Here, we present the results of our analysis of the association between different types of cataract and refractive errors.

There are some limitations to the present study, which should be considered when results are compared with those of other studies. The most important limitation is the cross-sectional design, which does not allow us to determine the causal effect of different cataract types on the incidence of refractive errors, and only the association of these two variables can be demonstrated.

Another limitation was the cut-off points used to define refractive errors and types of cataract. However, our main objective was to find any possible association between these two variables and not their own estimation in the population, and, thus, the definition seems to have a limited effect on the association.

Age-related changes in eyes affect visual function after 40 years of age. These problems can be easily solved by appropriate glasses, but lens opacities at older ages decrease visual acuity and bring about changes in the refractive status, which can lower the prescribed addition for presbyopia.

### Myopia and cataract

We found an association between myopia and cataract (regardless of the type). Our results showed that the risk of myopia is 1.81-times greater in subjects with nuclear cataract than in those free of cataract. Some studies^1^,^7^,^9^,^15^ and our previous report^12^ showed higher hyperopia with age. However, the increase in hyperopia was not as high as expected in the current study for subjects older than 60 years, probably due to the myopic effect of cataract.

Many other studies have shown the correlation between nuclear cataract and myopia.^1^,^4^–^7^,^16^ These studies suggest that nuclear cataract affects the density of the crystalline lens nucleus with an increased gradient index. These changes increase the refractive index and cause the image to form in front of the retina.

Large studies such as the Beaver Dam Eye Study and the Blue Mountains Eye Study point to the occurrence of cataract in different types of refractive errors, and state that myopia is a risk factor for cataract.^4^,^6^ Some studies have even shown that the degree of refractive errors is significantly correlated with increased lens opacity.^8^,^17^ Taken together, our data and other evidence^4^,^6^,^8^,^17^ indicate a correlation between myopia and cataract.

Although there is insufficient evidence to explain the occurrence of cataract with different types of refractive errors, we suggest two hypotheses. First, genetics play a significant role in high myopia, and, therefore, cataract in these people may also be a genetic predisposition. A second, more plausible explanation, is the fact that the vitreous thins earlier in myopes. This can facilitate the spread of oxygen to the posterior lens and result in lens opacity or cataract.^18^ Our results suggested that higher grades of nuclear cataract are associated with a myopic shift, and higher rates of myopia are seen [Table 2].

Because of the cross-sectional design of our study, we cannot detect the myopic shift in each individual as a result of increased severity of nuclear cataract. However, other studies support this association. In a Blue Mountains Eye Study report, Guzowski and colleagues^1^ showed that increased grades of nuclear cataract, especially grades 4 and 5, are associated with -0.34 D myopic shift. Gudmundsdottir and colleagues^7^ state this association as -0.65 D myopic shift 5 years after the diagnosis of a grade 2 or higher nuclear cataract.

The prevalence of myopia was significantly higher in PSC patients compared with those without cataract. Similar results have been reported in population-based studies such as the Beaver Dam Eye Study^6^ and Blue Mountains Eye Study.^19^ In two separate reports of the Blue Mountains Eye Study, the incidence and prevalence of PSC cataract in myopic patients were evaluated. Myopia was regarded as an independent factor in both reports. Although a causal relationship could not be established based on our results, other studies have reported an almost constant correlation between myopia and PSC.^6^,^19^

We found no significant correlation between cortical cataract and myopia. The peer-reviewed literature contains conflicting results on this issue. Xu and colleagues^20^ suggest that the correlation is reversed and the odds of myopia decrease with increasing severity of cortical cataract. Gudmundsdottir and colleagues^7^ studied the 5-year refractive changes in a population over 50 years of age and found no correlation. Kubo and colleagues^21^ confirmed Gudmundsdottir and colleagues^7^ ; however, the Blue Mountains Eye Study^2^ indicated an OR of 2.9 for high myopia in cases of cortical cataract.

### Hyperopia and cataract

Our results indicate that hyperopia was significantly lower in subjects with cataract compared with non-cataractous subjects. This is likely due to the increase of myopia in subjects with cataract. Additionally, no significant correlation was observed between hyperopia and different types of cataract in our study. However, the high percentage (77.78%) of hyperopia in subjects with cortical cataract is noteworthy. Although this correlation was not significant, a correlation between cortical cataract and hyperopia is probable considering the high percentage and the OR of 3.77. Only nine patients with cortical cataract participated in our study, which is considerably low for establishing a reliable statistical correlation. From the biologic perspective, cortical cataract tends to affect the composition of the lens, which leads to a decreased refractive index.

Studies on this issue have reached conflicting results. Some studies indicate that the correlation between cataract and hyperopia is weaker than with myopia, and some authors showed that hyperopia is correlated with nuclear cataract.^6^,^7^,^20^,^22^,^23^ For example, Fotedar and colleagues^9^ presented evidence of a significant shift toward hyperopia (0.26 D compared with baseline) among cases without nuclear cataract grades. In their study on elderly Chinese patients, Cheng and colleagues^24^ found an inverse correlation between nuclear cataract and hyperopia and higher degrees of hyperopia among cases with grade 1 and 2 nuclear cataracts compared with grades 5 and 6. There are other studies that confirm the correlation between hyperopia and lower grades of nuclear cataract.^9^,^15^,^24^ In our study, hyperopia seemed more prevalent among cases with grade 2 nuclear cataract compared with those without cataract, but this correlation was not seen with other grades of cataract. These observations are in agreement with previous findings of other studies concerning the correlation between low-grade nuclear cataract and hyperopia.^9^

## CONCLUSION

There is a correlation between cataract and refractive errors based on the results of this study. Individuals with nuclear cataract and PSC showed a significantly higher prevalence of myopia, while the prevalence of hyperopia was lower in those with cataract. High myopia was associated with higher grades of nuclear cataract. Although no significant correlation was found between cortical cataract and hyperopia, the higher percentage of hyperopia in these patients was noteworthy.

However, further studies are needed to evaluate this relationship.
